# Quantifying shape and ecology in avian pedal claws: The relationship between the bony core and keratinous sheath

**DOI:** 10.1002/ece3.5507

**Published:** 2019-09-30

**Authors:** Brandon P. Hedrick, Samantha A. Cordero, Lindsay E. Zanno, Christopher Noto, Peter Dodson

**Affiliations:** ^1^ Department of Cell Biology and Anatomy, School of Medicine Louisiana State University Health Sciences Center New Orleans LA USA; ^2^ Department of Earth Sciences University of Oxford Oxford UK; ^3^ Department of Earth and Environmental Sciences University of Pennsylvania Philadelphia PA USA; ^4^ North Carolina Museum of Natural Sciences Raleigh NC USA; ^5^ Department of Biological Sciences North Carolina State University Raleigh NC USA; ^6^ Department of Biological Sciences University of Wisconsin‐Parkside Kenosha WI USA; ^7^ Department of Biomedical Sciences, School of Veterinary Medicine University of Pennsylvania Philadelphia PA USA

**Keywords:** claw, individual variation, morphometrics, phylogenetic comparative methods

## Abstract

Terrestrial tetrapods use their claws to interact with their environments in a plethora of ways. Birds in particular have developed a diversity of claw shapes since they are often not bound to terrestrial locomotion and have heterogeneous body masses ranging several orders of magnitude. Numerous previous studies have hypothesized a connection between pedal claw shape and ecological mode in birds, yet have generated conflicting results, spanning from clear ecological groupings based on claw shape to a complete overlap of ecological modes. The majority of these studies have relied on traditional morphometric arc measurements of keratinous sheaths and have variably accounted for likely confounding factors such as body mass and phylogenetic relatedness. To better address the hypothesized relationship between ecology and claw shape in birds, we collected 580 radiographs allowing visualization of the bony core and keratinous sheath shape in 21 avian orders. Geometric morphometrics was used to quantify bony core and keratinous sheath shape and was compared to results using traditional arc measurements. Neither approach significantly separates bird claws into coarse ecological categories after integrating body size and phylogenetic relatedness; however, some separation between ecological groups is evident and we find a gradual shift from the claw shape of ground‐dwelling birds to those of predatory birds. Further, the bony claw core and keratinous sheath are significantly correlated, and the degree of functional integration does not differ across ecological groups. Therefore, it is likely possible to compare fossil bony cores with extant keratinous sheaths after applying corrections. Finally, traditional metrics and geometric morphometric shape are significantly, yet loosely correlated. Based on these results, future workers are encouraged to use geometric morphometric approaches to study claw geometry and account for confounding factors such as body size, phylogeny, and individual variation prior to predicting ecology in fossil taxa.

## Graphical abstract

Claw shape was quantified using geometric morphometrics and was compared to results using traditional arc measurements. Neither traditional nor geometric morphometrics are capable of significantly separating bird claws into coarse ecological categories after integrating body size and phylogenetic relatedness. Based on these results, future workers are encouraged to use geometric morphometric approaches to study claw geometry and account for confounding factors such as body size, phylogeny, and individual variation prior to predicting ecology in fossil taxa.

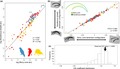

## 1 INTRODUCTION

Claws are important tools that vertebrates use to interact with their environments and are used for a variety of purposes, including locomotion, clinging to surfaces, food gathering, burrowing, and in inter‐ and intraspecific combat (Cartmill, 1974; Manning, Payne, Pennicott, Barrett, & Ennos, 2005; Reichman & Smith, 1990). Although the relationship between claw shape and ecological mode has been examined in birds (Feduccia, 1993) and lizards (D'Amore, Clulow, Doody, Rhind, & McHenry, 2018; Tulli, Abdala, & Cruz, 2011; Tulli, Cruz, Herrel, Vanhooydonck, & Abdala, 2009; Zani, 2000), this hypothesized relationship has most often been used for predicting the ecology of extinct taxa using the claw morphology of extant taxa (Birn‐Jeffery, Miller, Naish, Rayfield, & Hone, 2012; Feduccia, 1993; Fowler, Freedman, & Scannella, 2009; Fowler, Freedman, Scannella, & Kambic, 2011; Glen & Bennett, 2007). There have been comparatively few studies focusing explicitly on claw shape in extant birds, whether on the development and variability of claw morphology (Ethier, Kyle, Kyser, & Nocera, 2010) or the correlation between claw morphology and ecological mode (but see Csermely, Rossi, & Nasi, 2012; Csermely & Rossi, 2006; Pike & Maitland, 2004). Studies that have examined extant bird taxa find conflicting levels of correlation between claw shape and ecology, with different ecological modes often having large amounts of overlap in shape (Birn‐Jeffery et al., 2012; Pike & Maitland, 2004). Since birds are often not bound to terrestrial locomotion, their pedal claws have different constraints than obligate terrestrial taxa and are capable of taking on a wide spectrum of shapes, such as the long recurve found in many raptorial claws. Aves also has high body size disparity, ranging from the bee hummingbird (2.2 g) to the ostrich (111,000 g; Dunning, 1993), which may generate different constraints on pedal claw shape. Therefore, it would be expected that Aves would have high claw disparity, likely driven by different factors in different ecological groups, and that body mass would have a large impact on shape.

Previous workers examining claw morphometrics have adapted a version of the traditional morphometric arc length method first proposed by Peters and Görgner (1992) and Feduccia (1993) whereby claw shape is reduced to the angle of the claw arc. These methods have varied as to where the arc measurement was taken: on the dorsal surface of the claw (e.g., Pike & Maitland, 2004) or on the ventral surface of the claw (e.g., Feduccia, 1993), with many of these methods using the geometric properties of circles to reconstruct claw angles (reviewed by Tinius & Russell, 2017). However, in many bird species, claws do not inscribe a circle. Additionally, few studies have used phylogenetic comparative methods to incorporate the inter‐relatedness of representative taxa in statistical analyses (Felsenstein, 1985). Finally, the majority of vertebrate claws are composed of two basic components: the distal bony ungual and the keratinous sheath that envelops the bony core. Many studies have used the shape of the bony core in extinct taxa and the shape of the keratinous sheath in extant taxa interchangeably when making functional morphological assertions (but this has long been known to be problematic—Birn‐Jeffery et al., 2012). As a result, there is not yet a detailed understanding of how the bony core of the claw and the more friable keratinous sheath relate to one another and whether they can compatibly be compared.

Geometric morphometrics is a powerful technique for quantitatively analyzing shape data (Bookstein, 1991; Corti, 1993; Mitteroecker & Gunz, 2009; Slice, 2007; Zelditch, Swiderski, Sheets, & Fink, 2012), and has recently been used to approximate the shape of tetrapod claws and explore correlations between shape and ecology (D'Amore et al., 2018; Tinius & Russell, 2017). We expand on this recent work using a combination of geometric morphometric techniques and radiographs showing the inner bony core and outer keratinous sheath of a large sample of pedal digit III bird claws from across Aves. Using these data, we further test the findings of Tinius and Russell (2017) that the geometric morphometric approach is the best approximation of claw shape, by comparing a geometric morphometric approach with traditional morphometric arc measurements. Traditional morphometric and geometric morphometric data were extracted from both the bony core shape and keratinous sheath shape of each specimen to assess the following: (a) Is there a relationship between ecology and morphology in avian pedal claws after accounting for phylogenetic relatedness and body size (via a proxy)? (b) What is the range of individual variation in claw arc and shape? (c) Are the shapes of the bony claw core and keratinous sheath significantly correlated with one another or do they vary independently from one another? and (d) How closely do traditional morphometric and geometric morphometric data coincide? Although the relationship between claw shape and ecology in birds has been previously examined, this is the first study to look at claw shape comprehensively, incorporating keratinous sheath shape, bony core shape, and phylogenetic comparative methods with body size corrections.

## 2 MATERIALS AND METHODS

**Figure 1:**
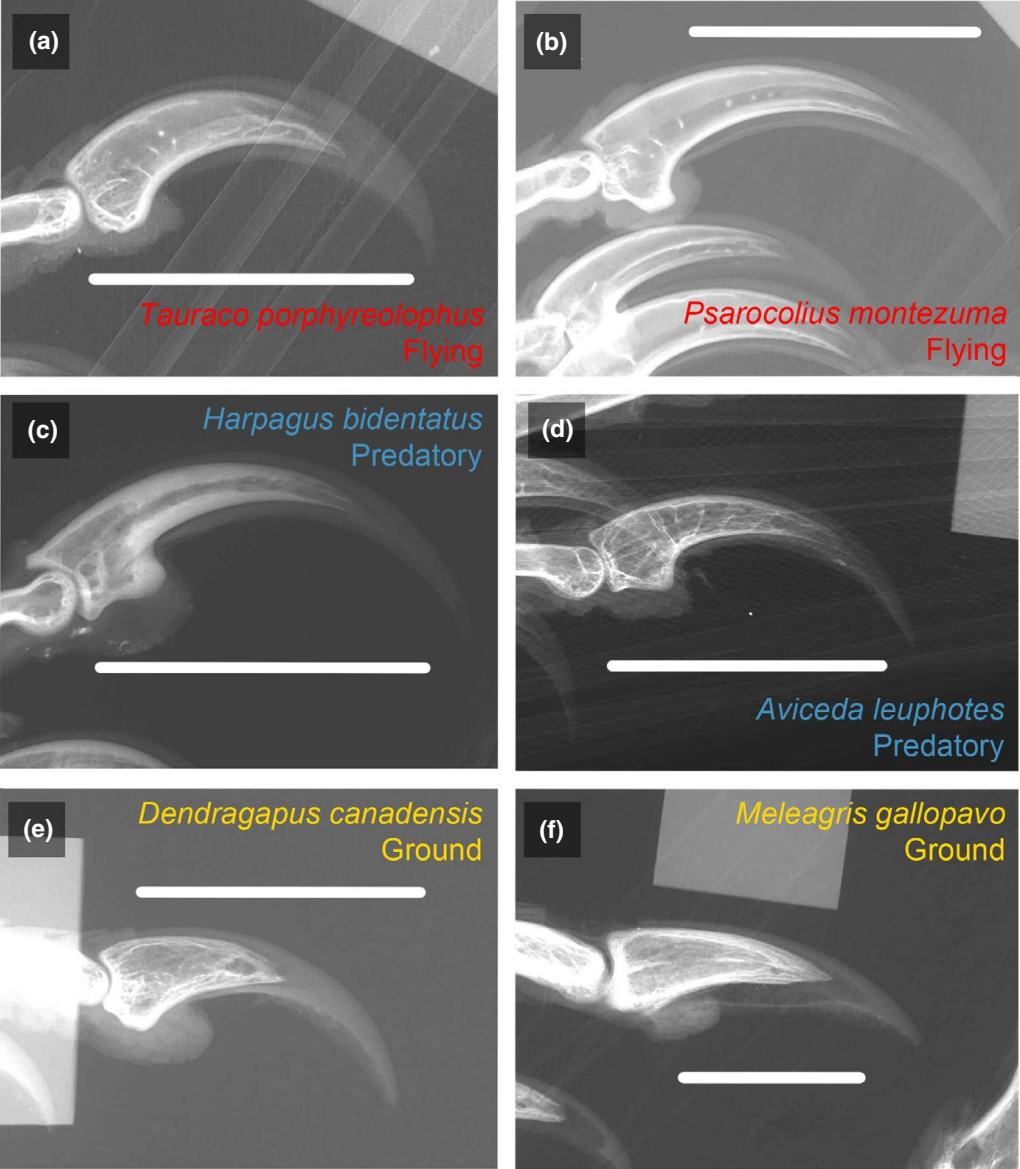
Representative third pedal unguals showing typical claws for each of the three ecological groups. Flying taxa: (a) *Tauraco porphyreolophus* (Purple‐crested Tauraco) and (b) *Psarocolius montezuma* (Black Oropendola); Predatory taxa: (c) *Harpagus bidentatus* (Double‐toothed Kite) and (d) *Aviceda leuphotes* (Pacific Baza); Cursorial taxa: (e) *Dendragapus canadensis* (Blue Grouse) and (f) *Meleagris gallopavo* (Wild Turkey)

Pedal digit III claws from 580 individuals of 145 species in 21 orders across the avian tree were x‐rayed (Figures 1 and S1). Specimens were selected to maximize phylogenetic coverage and allow for an assessment of individual variation. Digit III was used because it is the primary weight‐bearing toe (Glen & Bennett, 2007) and has often been used in previous studies of claw shape, affording comparability with past studies (Tinius & Russell, 2017). No obvious asymmetry was present between the left and right digit III of individual specimens, so left and right claws were radiographed interchangeably. They were positioned such that the sagittal plane of the claw was perpendicular to the x‐ray source to ensure that they were viewed in lateral view. We did not examine toes in addition to digit III due to documented significant interdigital variation within the same foot of individual specimens (Fowler et al., 2009). The specimens were radiographed using a Kodex Inc. Imagex 20i with Thermo Kevex x‐ray source (PXS10‐16w) and Varian Digital x‐ray detector. Images were taken at 40 kV and 266 μA with a spot size of 13 microns with 10.6 watts and 20 frames per radiograph. These radiographs allowed for visualization of both the bony core and keratinous sheath of each specimen. Some species were sampled at particularly high rates to assess intraspecific variation in claw shape (e.g., *Tinamus major*, *n* = 25). To facilitate phylogenetic comparative analyses, species means were taken for the 145 species for both traditional and geometric morphometric measures (Tinius & Russell, 2017). The maximum credibility phylogeny for extant birds generated by Jetz, Thomas, Joy, Hartmann, and Mooers (2012) was pruned to include only the species sampled in this analysis and was used for phylogenetic comparative analyses (Figure S1).

Taxa were then split into three ecological groups (predominantly predatory, predominantly ground‐dwelling, and flying generalists) to assess how claw shape related to ecology. Birds of prey (e.g., Accipitriformes, Strigiformes, Falconiformes) were classified as predominantly predatory birds given the unique ways that they use their claws in prey capture (Brown & Amadon, 1968; Csermely et al., 2012; Csermely & Rossi, 2006; Del Hoyo, Hoyo, Elliott, & Sargatal, 1992; Johnsgard, 1990). Birds were considered predominantly ground‐dwelling birds if they spend the majority of their time, or all of their time, on the ground (e.g., ratites, bustards, some Galliformes; Del Hoyo et al., 1992). Flying generalists included birds that use flight as their primary mode of locomotion and included a wide range of groups and flight styles (e.g., Passeriformes, Apodiformes, Psittaciformes, Coraciiformes; Del Hoyo et al., 1992). To better balance sample sizes within ecological groups, flying generalists were not further split into climbing and perching birds as has been done by previous workers (Glen & Bennett, 2007; Pike & Maitland, 2004). This is further justified given recent work showing a lack of clear separation between those two groups (Tinius & Russell, 2017).

**Figure 2:**
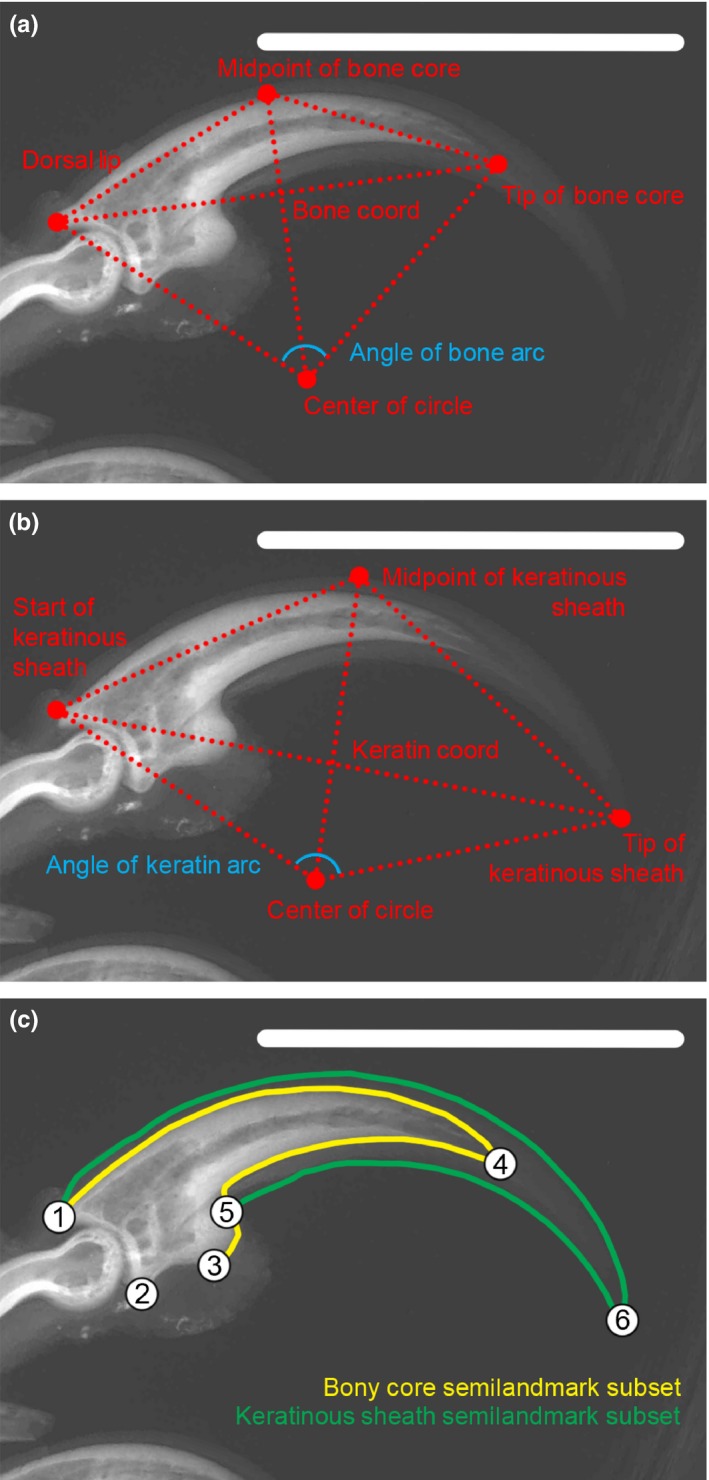
Traditional arc measurements taken for the (a) bony core and (b) keratinous sheath. (c) Landmark configuration with numbered landmarks and semilandmark curves for the bony core (yellow) and keratinous sheath (green). Landmark definitions in Table 1

Three traditional morphometric measures were taken from claw radiographs: (a) the ratio of length of the bony core to length of keratinous sheath; (b) the dorsal arc of the bony core (Figure 2a); and (c) the dorsal arc of the keratinous sheath (Figure 2b). These measurements were taken from radiographs in ImageJ (Schneider, Rasband, & Eliceiri, 2012) following the general scheme set forth by Pike and Maitland (2004). The ventral arc of the claw was not calculated, given that many claws have a ventral constriction near the claw tip (Pike & Maitland, 2004) and that the dorsal arc gives a better estimate of claw curvature than the ventral arc (Tinius & Russell, 2017). Note that these traditional methods assume that both the arcs of the bony core and keratinous sheath inscribe a circle. Seven points were located on each claw: the tip of the keratinous sheath, the tip of the bony core, the midpoint of the crescent‐shaped articulation surface with the penultimate phalanx, the dorsal lip of the bony core, the dorsal lip of the keratinous sheath, and approximate midpoints along the arcs of the bony core and keratinous sheath. These points were used to calculate the center of the circle that the bony core and keratinous sheaths inscribe, which were then used to calculate arcs. The lengths of the bony core and keratinous sheath were calculated using an arc from the midpoint of the crescent‐shaped articulation with the penultimate phalanx and the tip of the bony core and keratinous sheath, respectively. These values were then used to generate a ratio of bony core to keratinous sheath length for each claw (for detailed information on measurement protocols, see the Appendix S1).

For geometric morphometric analyses, six landmarks and 77 semilandmarks were digitized onto the radiographs (Figure 2c; Table 1) using the tpsDig2 software (Rohlf, 2006). Semilandmarks (sLMs) were split into four curves: along the dorsal keratinous sheath (28 sLMs), the dorsal bony core (13 sLMs), the ventral bony core (18 sLMs), and the ventral keratinous sheath (18 sLMs). The number of sLMs was selected so as to adequately represent the curves without saturating the curve with landmarks. Semilandmarks were slid according to the bending energy criterion (Perez, Bernal, & Gonzalez, 2006). The goal underlying landmark selection was to capture both the shape of the bony core and the keratinous sheath. The landmark data were imported into the R package *geomorph* (Adams & Otárola‐Castillo, 2013) and subjected to Generalized Procrustes Analysis (GPA).

**Table 1:** Landmarks, semilandmarks, and landmark definitions for geometric morphometric data

Landmark number	Type	Definition	Subset
1	II	Apex of the curve of the proximodorsal convexity at the articulation of the ungual with phalanx III	Bony core
2	II	Apex of the curve of the proximoventral convexity at the articulation of the ungual with phalanx III	Bony core
3	II	Apex of the curve of the proximoventral‐most extension of the flexor tubercle	Bony core
4	II	Distal tip of ungual	Bony core
5	I	Point where the ventral keratinous sheath meets the bony ungual	Keratinous sheath
6	II	Distal tip of keratinous sheath	Keratinous sheath
SL‐1	Semi	From LM 1–4	Bony core
SL‐2	Semi	From LM 3–4	Bony core
SL‐3	Semi	From LM 1–6	Keratinous sheath
SL‐4	Semi	From LM 5–6	Keratinous sheath

Note

Bookstein's topology of landmarks are noted for each landmark. Further, each landmark is divided into either the bony core or keratinous sheath subset for functional integration and modularity analyses.

### 2.1 Ecological signal accounting for impacts of phylogeny and size

To assess the relationship between traditional claw metrics and ecological groups quantitatively, phylogenetic generalized least squares (PGLS) regressions using maximum likelihood estimates of Pagel's lambda (Pagel, 1999) were run in the *nlme* package in R (Pinheiro, Bates, DebRoy, & Sarkar, 2018; R Core Team, 2018). Therefore, these analyses do not assume Brownian motion or a star phylogeny. Pairwise comparisons were performed on species means for each traditional metric using *phytools* in R (Revell, 2012). All PGLS models included size as a factor, as some previous studies have found a significant impact of size on claw arc metrics (Birn‐Jeffery et al., 2012; Csermely et al., 2012; Pike & Maitland, 2004). While body mass is commonly used as a size metric, museum specimens do not usually have body mass recorded and only 89% of the included taxa had known body masses in the literature (Dunning, 2007). Further, sex was unknown for some of the specimens included in this study and sexual size dimorphism is large in many of the sampled species (e.g., *Meleagris gallopavo*). Therefore, taking the average of male and female body masses would likely have led to poor estimates of actual body mass for the specimens. As an alternative, claw centroid size—the square root of the sum of squared interlandmark distances—was used as the body mass metric. Previous studies have found that the size of pedal digit III claws and body masses are tightly correlated in birds regardless of ecological mode over a wide range of body masses (Pike & Maitland, 2004). Future studies are encouraged to examine the correlation between body mass of individual museum specimens and claw centroid size, but unfortunately these data were not available for our sample.

To evaluate geometric morphometric data, a PCA was run on species means to distinguish between taxon trends in morphospace in *geomorph* (Adams & Otárola‐Castillo, 2013) in R (R Core Team, 2018). The impacts of phylogeny were assessed using the multivariate version of Blomberg's *K* statistic (Adams, 2014; Blomberg, Garland, & Ives, 2003). The degree of allometric signal in the data was determined by testing for a correlation using a Procrustes ANOVA between the common allometric component and log‐transformed centroid size of the claws (Mitteroecker, Gunz, Bernhard, Schaefer, & Bookstein, 2004). Given a significant allometric signal, a phylogenetic Procrustes ANOVA (Goodall, 1991) was run testing the relationship between shape, size, and ecological group. Finally, differences in levels of disparity were evaluated using Procrustes variance as the disparity metric (Zelditch et al., 2012) using 999 permutations to calculate significance.

### 2.2 Individual variation

The amount of variance and range of values for each traditional metric was calculated for the entire dataset and for four species which had the largest sample sizes among the data to assess individual variation surrounding species means (*Milvus migrans—*Black kite, Accipitriformes, *n* = 19; *Dendragapus canadensis*—Spruce grouse, Galliformes, *n* = 20; *Puffinus griseus*—Sooty shearwater, Procellariiformes, *n* = 21; *Tinamus major—*Great tinamou, Tinamiformes, *n* = 25). For geometric morphometric data, a principal component analysis (PCA) was run on all data (*n* = 580) highlighting these four species to visualize the impact of individual variation on morphospace occupation.

### 2.3 Relationship between the bony core and keratinous sheath

PGLS models were performed on species means with log‐transformed centroid size as a covariate to test whether significant correlations between the log‐transformed bony core and log‐transformed keratinous sheath arcs were present. The *R* ^2^ coefficient was used to assess the amount of variance of the bony core arc that explained the keratinous sheath arc. Then, a phylogenetic ANOVA with pairwise comparisons of the residuals from the above PGLS and ecological group was used to determine whether this relationship was different among the three hypothesized ecological groups. Finally, a phylogenetic paired *t* test was run comparing the bony core arc measurements with the keratinous sheath arc measurements to test if the bony core arcs and keratinous sheath arcs were statistically different from one another.

For geometric morphometric data, claw landmarks were placed into two separate subsets following GPA. Landmarks 1, 2, 3, 4, and 7–37 were assigned to the bony core subset and landmarks 5, 6, and 38–83 were assigned to the keratinous sheath subset (Figure 2c). Hypotheses of modularity and functional integration were then tested for species means accounting for the impacts of phylogeny under a Brownian motion model of evolution in *geomorph* (Adams, 2016; Adams & Collyer, 2016; Adams & Felice, 2014). Note that these analyses are testing for functional modularity and integration and are not based on hypothesized developmental modules. The aim was to determine whether the keratinous sheath and bony core function as a single unit or as two separate modules to see if it is possible to conflate these two types of data in analyses including fossils for which the keratinous sheath is not known. The covariance ratio (CR) coefficient was calculated from the data and then compared to a null distribution of CR values based on landmarks being randomly assigned to the two landmark subsets for 999 iterations. When the observed CR coefficient is significantly lower than the null distribution, the hypothesis of modularity is supported. Functional integration was evaluated using phylogenetic partial least squares (PLS) analysis. Significance was determined by randomly permuting landmarks in the two landmark subsets for 999 iterations. Differences in degrees of functional integration between ecological groups were then compared using the compare.pls function in *geomorph*.

### 2.4 Comparison of traditional and geometric morphometric methods

Finally, two‐block PLS analysis was employed to assess the degree of similarity between the traditional and geometric morphometric data. The three traditional morphometric measures (ratio of bony core length to keratinous sheath length, log‐transformed bony core arc, log‐transformed keratinous sheath arc) were combined into a single block and the geometric morphometric shape data were combined into the second block. As above, phylogenetic two‐block PLS analyses were run using species means and 999 iterations in *geomorph*. Further, a nonphylogenetically corrected PLS analysis was done between traditional metrics and geometric morphometric data using individuals (*n* = 580) rather than species means.

## 3 RESULTS

### 3.1 Ecological signal accounting for impacts of phylogeny and size

Phylogenetic ANOVAs revealed that ecological groups are not significantly correlated with the length ratio (*p* = .272), log‐transformed bony core arc (*p* = .489), or log‐transformed keratinous sheath arc (*p* = .314) after accounting for size. Similar to the traditional metrics, geometric morphometric data did not show a significant difference in claw shape across groups in spite of apparent groupings in PCA (ground vs. predatory, *p* = .692; ground vs. flying, *p* = .506; flying vs. predatory, *p* = .981; Table S3). Although the phylogenetic ANOVAs incorporated maximum likelihood estimates of lambda, the majority of predatory birds came from the Accipitriformes and this may have affected significances when incorporating phylogeny.

**Figure 3:**
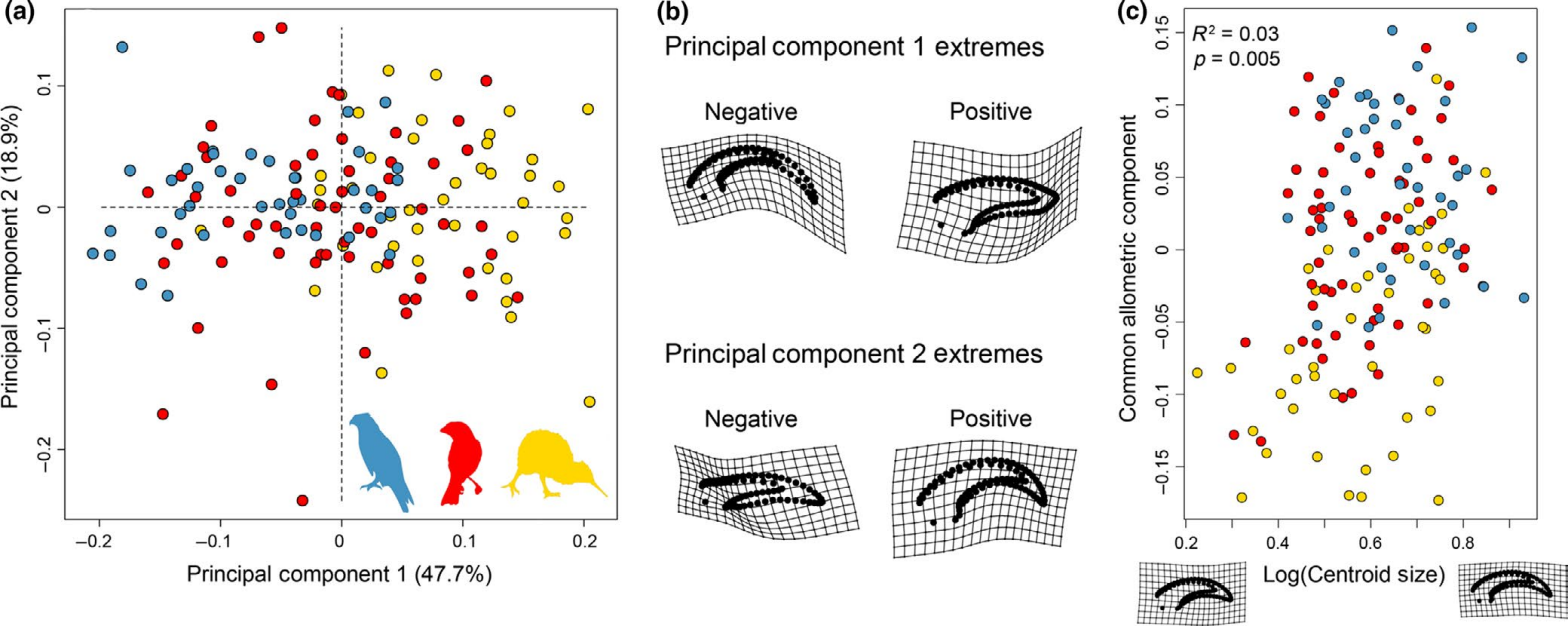
Geometric morphometric claw shape data. (a) Principal component analysis of total claw shape showing separation between predatory and ground birds with flying birds spreading across morphospace. Blue = predatory, red = flying, yellow = ground. (b) Thin‐plate spline (TPS) representations of the positive and negative extremes of PC1 and PC2. (c) Allometric analysis of the common allometric component of shape and log‐transformed centroid size. TPS grids show representations of small (left) and large (right) claw shape

For geometric morphometric means data, principal component 1 (PC1) summarized 47.7% of total variance and PC2 summarized 18.9% of total variance (Table S4). Although PC1 revealed separation between ground‐dwelling and predatory taxa (Figure 3a), flying generalists ranged broadly across morphospace. Taxa on the positive end of PC1 had blunt short claws in which the bony core to keratinous sheath length ratio was high. These shapes were similar to those of ground‐dwelling taxa (Figure 3b). Taxa on the negative end of PC1 had claws with a strong recurve similar to predatory taxa with a bony core to keratinous sheath length ratio closer to 0.70. PC2 did not separate the three ecological groups. The consensus shape for the positive end of the PC2 axis had a slight recurve and a high bony core to keratinous sheath ratio. The negative end of PC2 was characterized by a flattened, elongated claw. The geometric morphometric data were significantly correlated with phylogeny (*K*_mult_ = 0.155, *p* < .001) and allometry (Figure 3c), albeit with a low percent of shape variance explained by allometry (*R* ^2^ = .03). Procrustes variance was roughly the same for all three groups (flying generalists = 0.71, ground dwellers = 0.66, predatory = 0.72), with no groups having significantly different levels of disparity.

### 3.2 Individual variation

**Figure 4:**
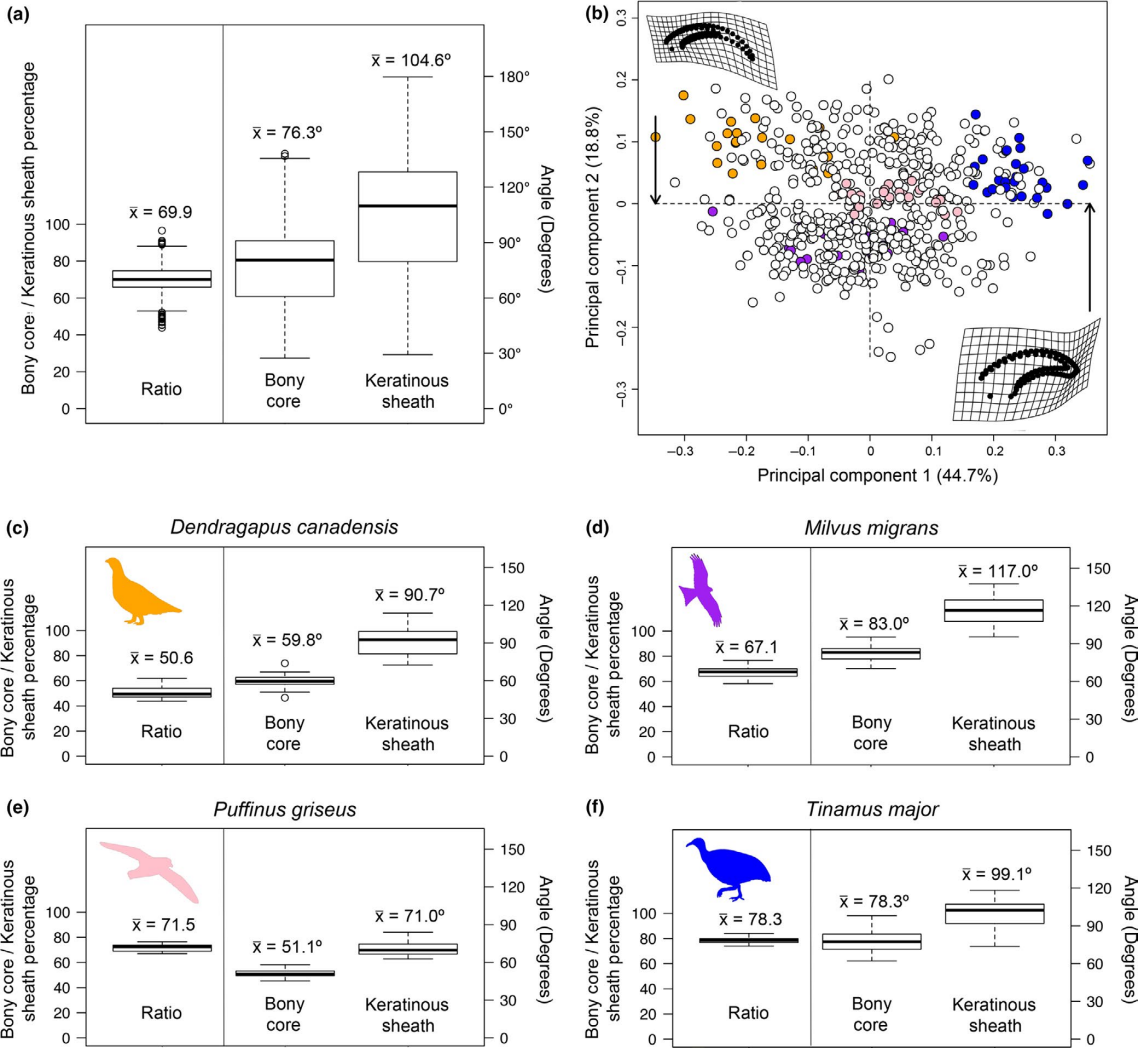
Range of variation for (a) traditional measurements and (b) geometric morphometric data of the combined keratinous sheath and bony core. Orange*—Dendragapus canadensis* (*n* = 20); Purple—*Milvus migrans* (*n* = 19); Pink—*Puffinus griseus* (*n* = 21); Dark blue—*Tinamus major* (*n* = 25). Variation in traditional morphometric metrics for (c) *Dendragapus canadensis*, (d) *Milvus migrans*, (e) *Puffinus griseus*, and (f) *Tinamus major*. For traditional metrics, the variation of the ratio of the bony core to keratinous sheath is displayed to the left of the vertical line using the left x‐axis and the variation of the angle of the bony core and keratinous sheath are displayed to the right of the vertical line using the right x‐axis for all taxa together (a) and individual taxa (c–f)

All three traditional morphometric measures had relatively low variance across all taxa. The 95% confidence intervals for variations in the ratio of the length of the bony core to the length of the keratinous sheath length ratio ranged from 0.693 to 0.705, the bony core arc ranged from 74.6° to 77.9°, and the keratinous sheath arc ranged from 102.2° to 107° (Figure 4a). When assessing intraspecific variance, the four highly sampled taxa had confidence intervals that were a maximum of 2%–4.1% wide for the length ratio, 2.64–7.08° wide for the bony core arc, and 4.66–10.3° wide for the keratinous sheath arc (Figure 4c–f; Table S1). While these confidence intervals were not large in absolute terms, they suggest that individuals of the same species may be more different from one another than individuals of different, related species. The geometric morphometric data demonstrate that each of these four taxa clusters intraspecifically in PCA, but that each taxon ranges across morphospace such that the intraspecific variation is often larger than interspecific variation (Figure 4b; Table S2). Both traditional and geometric morphometric data demonstrate substantial intraspecific variation in claw shape.

### 3.3 Relationship between the bony core and keratinous sheath

**Figure 5:**
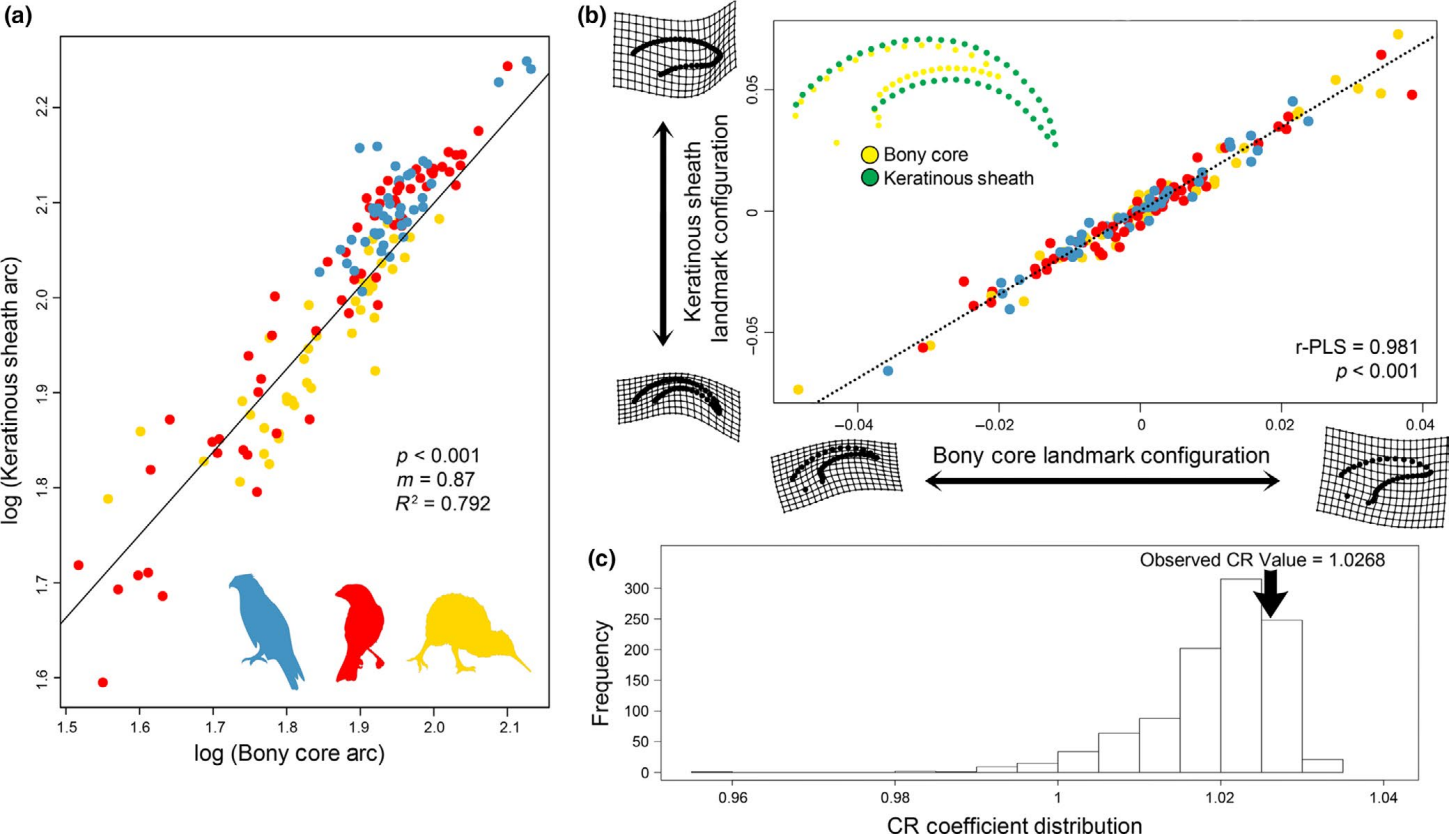
Measures of integration between the bony core and keratinous sheath for both (a) traditional morphometric data using phylogenetic general least squares regression (blue = predatory, red = flying, yellow = ground) and (b) geometric morphometric data using two‐block partial least squares analysis. TPS grids show differences in shape along each shape block. (c) Assessment of modularity showing the observed covariance ratio (CR) against a null distribution. The observed CR is not significantly lower than the distribution and so modularity is not supported

A PGLS of log‐transformed keratinous sheath arc and log‐transformed bony core arc data including centroid size as a factor had a significant association (*λ* = 0.702, *m* = 0.873, *p* < .001) with a substantial amount of total variance of the bony core arc explaining the keratinous sheath arc (*R* ^2^ = .792; Figure 5a). Residuals of this PGLS and ecological group did not reveal a significant association (*p* = .185) suggesting that different ecologies did not have differing trajectories between their bony core arc and keratinous sheath arc. A *t* test did show that the log‐transformed bony core arc and log‐transformed keratinous sheath arc were significantly different from one another (*p* < .001) with the keratinous sheath having a greater angle than the bony core.

Using geometric morphometric data, the phylogenetically informed PLS analysis supported significant functional integration between the bony core and keratinous sheath (r‐PLS = 0.981, *p* < .001). This was additionally supported by a PLS analysis on individuals rather than species means (Figure S2). Taxa with recurved bony cores had recurved keratinous sheaths and taxa with flattened, short bony cores had flattened, short keratinous sheaths (Figure 5b). Further, the phylogenetically informed analysis of modularity did not support the bony core and keratinous sheath as separate modules (CR = 1.02, *p* = .845; Figure 5c). Comparison of integration levels did not suggest that any ecological group had a greater degree of functional integration than any other group (Table S3).

### 3.4 Comparison of traditional and geometric morphometric methods

**Figure 6:**
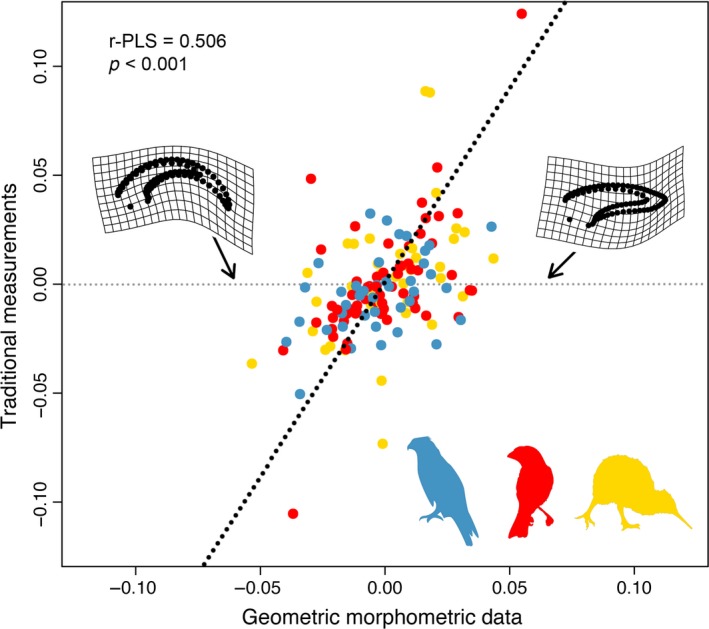
Two‐block partial least squares analysis of geometric morphometric data against traditional morphometric data. Inset TPS grids show shapes at the positive and negative ends of the geometric morphometric block. Blue = predatory, red = flying, yellow = ground

A phylogenetic PLS of traditional metrics and geometric morphometric shape data had a significant, but loose correlation (r‐PLS = 0.506, *p* < .001, Figure 6). This suggests that the two types of data summarize claw shape in somewhat complementary ways, but that the data generated through traditional morphometrics and geometric morphometrics do not show a strong degree of correlation.

## 4 DISCUSSION

Avian claw shape has immense variability (Figure 1). Claw arc measurements were first used in the early 1990s, with the goal of predicting the ecology of fossil bird taxa based on the relationship between claw shape and ecology in extant birds (Feduccia, 1993; Peters & Görgner, 1992). Since that pioneering work, some studies have found a strong correlation between claw shape and ecology in birds (Csermely et al., 2012; Glen & Bennett, 2007) while others have not (Birn‐Jeffery et al., 2012; Pike & Maitland, 2004). These conflicting results are exacerbated by the lack of overlap in taxa and methodologies across studies (Birn‐Jeffery et al., 2012). Additionally, many paleobiologists have used keratinous sheath shape in extant taxa to attempt to reconstruct ecological mode in extinct taxa using bony core shape, which have thus far been assumed to be complementary. After incorporating phylogeny and body size, we found that neither traditional nor geometric morphometrics recovered significant differences in claw shape between ecological categories. The bony core and keratinous sheath are significantly functionally integrated and the degree of functional integration does not differ across ecological groups, but the bony core shape and keratinous sheath shape are significantly different and cannot be compared without corrections.

Although body mass was found to be significantly correlated with claw shape, we support previous studies in asserting that body size is not a substantive predictor of claw shape (Birn‐Jeffery et al., 2012; Pike & Maitland, 2004) given that only 3% of total variance is explained by size (Figure 3b). Tinius and Russell (2017) found a significant relationship between body mass (Dunning, 2007) and claw geometry and shape, but suggest that this is related to size differences in each ecological cluster (e.g., ground‐dwelling birds are typically larger than perching birds). The relationship between body mass and claw arc has been shown to be complex, wherein the relationship likely varies within ecological categories (Birn‐Jeffery et al., 2012; Pike & Maitland, 2004; Tinius & Russell, 2017). For example, claw angle increases with body mass in predatory and climbing birds, but decreases with body mass in ground birds, and is not correlated with body mass in perching birds (Pike & Maitland, 2004). Therefore, it is important to include a size correlate in models even if the overall variation explained by size is small. Studies that do not include size as a predictor variable may introduce confounding effects.

Similar to body size, phylogenetic relatedness is a likely confounding effect in any comparative study (Felsenstein, 1985) and differing relationships between claw shape and ecology in previous studies may be due to a lack of the consistent application of phylogenetic comparative methods. A strong phylogenetic signal was uncovered for claw shape whether it was derived using traditional or geometric morphometrics, demonstrating the importance of using phylogenetic comparative methods when examining claw shape. Recently, Cobb and Sellers (2019 [Preprint]) have argued that phylogenetic comparative methods cannot be used for comparative studies of birds, citing recent conflicting bird trees. However, there are multiple avian trees representing all known extant bird taxa, which largely agree (Jetz et al., 2012; Prum et al., 2015). Therefore, birds represent one of the best vertebrate groups on which to apply phylogenetic comparative methods. Previous claw studies have found that morphological trends follow family level groupings in the absence of phylogenetic comparative methods (Fowler et al., 2009) and that the implementation of independent contrasts largely eliminates the significant relationship between claw geometry and behavior (Birn‐Jeffery et al., 2012). Phylogenetic comparative methods must be employed when assessing the relationship between claw shape and ecology in birds due to substantial ecological convergence in groups separated by long branch lengths.

Although there were no significant associations between ecology and shape, the PCA of geometric morphometric shape data did show some separation between ecological groups (as found by Tinius & Russell, 2017) when using geometric morphometrics, especially between predatory and ground birds. This is likely because predatory birds use their highly recurved claws in prey capture while ground‐dwelling birds typically have flat claws to ensure that the claws do not interfere with locomotor ability. The generalist flying category was spread widely across morphospace, invading the predatory and ground bird regions of morphospace (Figure 3a). It is therefore possible that our groupings were too broad to detect ecological differences, especially because members of our groups may have used their claws in noncomplementary ways. For example, both Strigiformes and Accipitriformes were placed in the predatory category, but previous studies have found that they strongly separate in claw shape morphospace due to different prey capture techniques (Csermely et al., 2012). Tinius and Russell (2017) argue that using preordained ecological groups may lead to a lack of significant differences because claw shape is organized along a spectrum rather than in discrete clusters. For example, many workers have examined perchers and climbers as separate categories, but Tinius and Russell (2017) found them to unite in a single cluster. Indeed, we find a gradual shift from claws of ground‐dwelling birds to those of predatory birds along PC1 (Figure 3a) rather than distinct clusters. The groupings used in this analysis were general in an attempt to detect broad trends in the dataset and were based on groupings used in previous analyses. However, finer‐scale analyses with a larger dataset of taxa may be capable of distinguishing significant groupings among birds. We concur with Tinius and Russell (2017) that it is likely that geometric morphometrics may discriminate claw shape across ecological groups better than traditional morphometrics, especially considering that these two data types are only loosely correlated with one another (Figure 6). The only detriment to performing geometric morphometrics in the analysis of claw shape in birds is the increased time required to collect the data (Tinius & Russell, 2017). Further, the shape of bird claws is strongly phylogenetically structured in spite of substantial convergence across the phylogeny and incorporating phylogenetic comparative methods in assessments of claw shape is critical to obtaining reliable results.

Additionally, there was a previously unrecognized confounding factor present in the data, high intraspecific variation. Some previous studies used up to six specimens per species when examining claw shape (Birn‐Jeffery et al., 2012) while some other studies have used a single individual per species (Csermely et al., 2012). When we examined several species that had high sample sizes in our dataset (*n* > 18) very high intraspecific variation was observed, whether using traditional measurements or geometric morphometric analyses (Figure 4). Plotting four species with large sample sizes in morphospace showed some within‐species clustering, but the spread of each cluster ranged quite widely, often taking up a large portion of occupied morphospace (Figure 4b). Ethier et al. (2010) warned workers not to use a single bird species in studies of claw development or morphology as a result of variable rates of claw growth due to fluctuating energy demands such as migration and breeding. Cobb and Sellers (2019 [Preprint]) found that the left and right claws of the same fossil taxon could be categorized in different ecological categories. Although this may also be related to taphonomy (Hedrick & Dodson, 2013; Hedrick, Schachner, Rivera, Dodson, & Pierce, 2019), no study has thus far looked at within‐individual variation in claw shape in extant or fossil birds. It is likely that intraspecific variation is an additional, previously unappreciated factor that may have led to discrepancies in results in prior studies.

The size and shape of the bony core and keratinous sheath of avian claws has previously been considered to be similar (Clark, 1936; Ethier et al., 2010), but this relationship had not been tested. We found bony core and keratinous sheath to be significantly and strongly correlated, suggesting they act as a functional unit, using both traditional morphometric (Figure 5a) and geometric morphometric approaches (Figure 5b,c). This is important to establish because there is a large body of work that predicts ecology in extinct taxa by comparing the bony cores of extinct taxa to the keratinous sheaths of extant taxa. Glen and Bennett (2007) presented the first estimation of the keratinous sheath arc from the bony core arc using radiographs, but they did not present the strength of the correlation, only the conversion factor. Recently Cobb and Sellers (2019 [Preprint]) used radiographs to determine whether the bony ungual or keratinous sheath was a better predictor of avian ecology, but they did not test for the correlation between the two data types. We found that the log‐transformed bony core arc explains 79.2% of the variation in the log‐transformed keratinous sheath arc when incorporating phylogeny and body size, suggesting a relatively tight correlation (Figure 5a). The geometric morphometric data had an even stronger correlation between the bony core and keratinous sheath (Figure 5b,c). These data suggest that predicting keratinous sheath shape from bony core shape is feasible and is better done using geometric morphometrics than traditional morphometrics. However, the bony core shape and keratinous sheath shape are statistically different from one another in spite of being correlated and therefore it is inappropriate to directly compare the bony core of fossil claws with the keratinous sheath of extant taxa without a conversion factor.

These results have strong implications for paleobiologists attempting to reconstruct fossil bird or nonavian dinosaur ecology using bony core shape. Except in cases of excellent preservation, the bony core is the only structure that is preserved and the keratinous sheath is lost or degraded. Although the keratinous sheath and bony core are strongly correlated with one another and do not function as separate modules (Figure 5), the arc of the bony core is typically quite different from that of the keratinous sheath. Based on arc measurements, the keratinous sheath generally has a greater arc than that of the bony core, although there is substantial variation (Figure 5a). Similarly, although more curved bony cores have highly curved keratinous sheaths, for a given amount of shape change in the bony core, there is substantially more shape change in the keratinous sheath (Figure 5b). These two types of data cannot reliably be conflated, but it would likely be possible to use extant data to reconstruct the shape of the keratinous sheath in fossils from bony core shape with additional study, leading to more reliable reconstructions of the shape of fossil claws.

As first noted by Pike and Maitland (2004), one limitation of this study and all previous studies is that these analyses were all run on two‐dimensional representations of three‐dimensional structures. Predatory birds often have conical, tapering claws whereas climbing birds have laterally compressed claws with sharp distal ends (Peters & Görgner, 1992; Pike & Maitland, 2004; Richardson, 1942; Yalden, 1985). Throughout the course of this work, we noticed numerous claws that were mediolaterally asymmetric, such as those of some woodpecker and parrot species. Although this has yet to be reported in a quantitative framework, it is likely related to function. This information is completely lost when quantifying shape using photographs or radiographs and future claw shape studies using CT will be necessary to better categorize birds into distinct ecological categories.

## 5 CONCLUSION

After accounting for body size and phylogenetic relatedness, neither traditional morphometrics nor geometric morphometrics are capable of significantly separating birds into a priori ecological categories. However, ordination analyses do demonstrate some separation of claw groups in morphospace when using geometric morphometrics, suggesting that geometric morphometrics leads to increased ecological classification in comparison with traditional morphometric methods in claws, as it does for a wide range of structures in many different groups (e.g., Hedrick & Dumont, 2018; Schmieder, Benítez, Borissov, & Fruciano, 2015; Tinius & Russell, 2017). We find support for the assertion that claw shape varies along a spectrum and the relationship between ecological patterns and claw shape is complex (Tinius & Russell, 2017). Further, we documented high intraspecific variation in claw shape using both geometric morphometric and traditional measurements and caution against using single specimens in studies of avian claw shape. Although there has been a large amount of previous work on claw shape, differing methods of data collection and analysis have precluded a consensus on how avian ecology impacts claw shape. We advocate for geometric morphometrics of claw radiographs or CT scans as a promising new method for characterizing claw shape and strongly suggest that future studies incorporate confounding factors such as body size, phylogeny, and individual variation before assessing ecology in extinct taxa.

## CONFLICT OF INTEREST

The authors declare no conflict of interest.

## AUTHOR CONTRIBUTIONS

BPH, SAC, LEZ, CN, and PD conceived of the study. SAC collected the data and landmarked the claws. BPH and SAC analyzed the data. BPH, SAC, LEZ, CN, and PD wrote the manuscript.

## Data Availability

Original data including radiographs and supplemental information are archived into the public repository Dryad https://doi.org/10.5061/dryad.k492m37.
