# Supplementary figures and images for: Quantifying shape and ecology in avian pedal claws: The relationship between the bony core and keratinous sheath
Source: Ecol Evol. 2019 Sep 30;9(20):11545–56. doi: 10.1002/ece3.5507 (PMC6822041; doi:10.1002/ece3.5507)

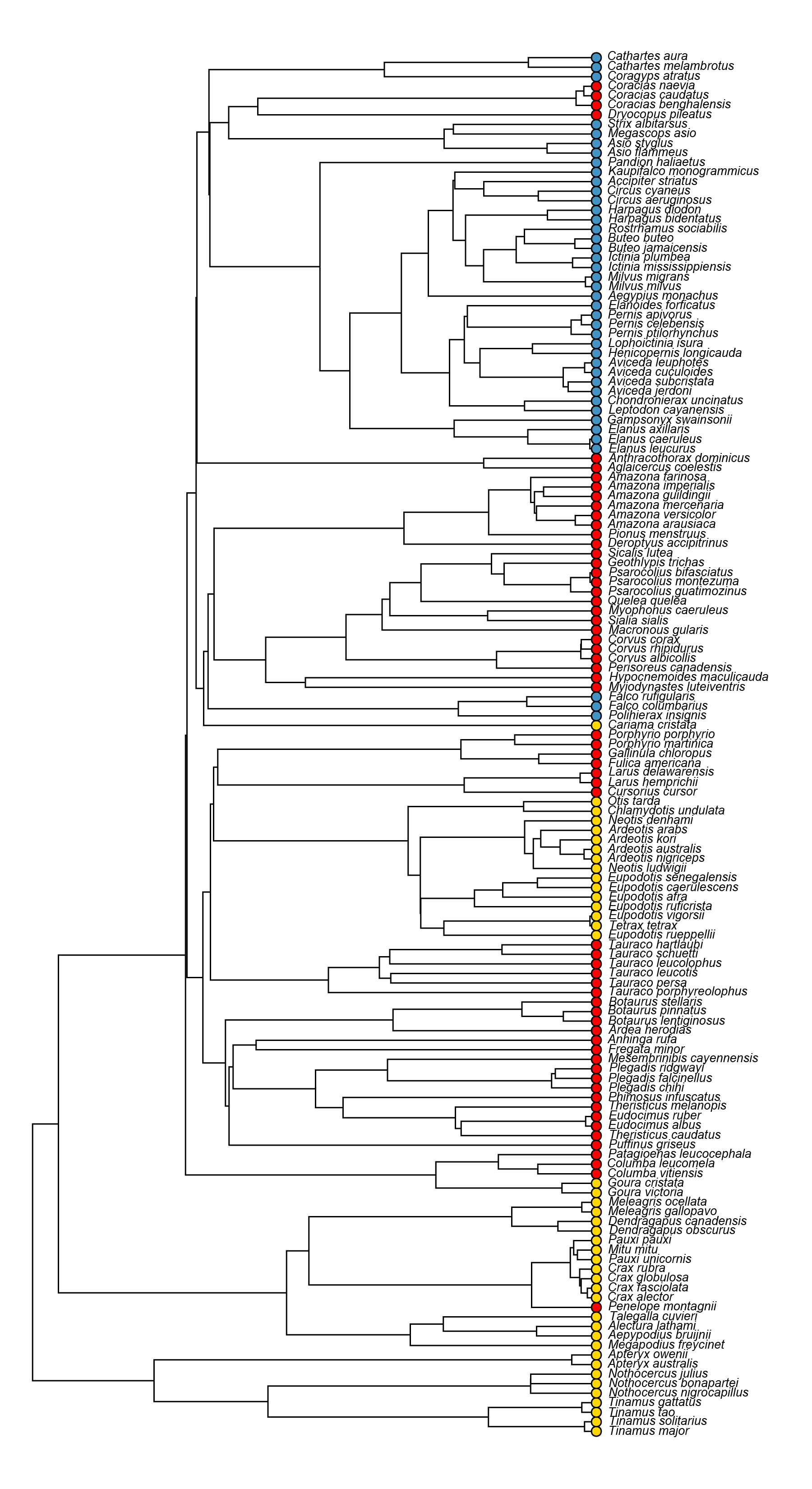

Supplement: Supplementary file 1 [file ECE3-9-11545-s001.tif]

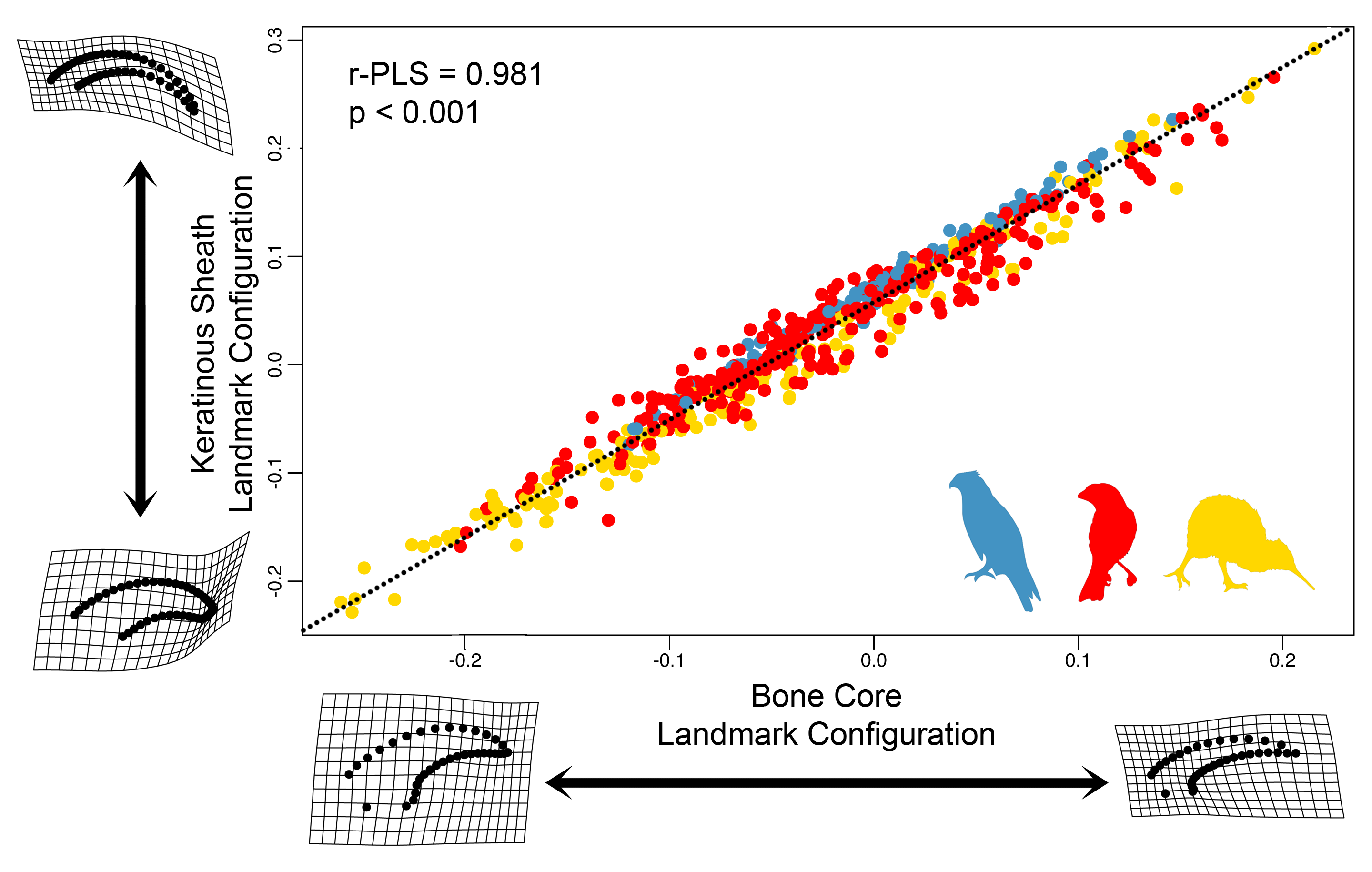

Supplement: Supplementary file 2 [file ECE3-9-11545-s002.tif]
