# Supplementary material for: Quantifying shape and ecology in avian pedal claws: The relationship between the bony core and keratinous sheath
Source: Ecol Evol. 2019 Sep 30;9(20):11545–56. doi: 10.1002/ece3.5507 (PMC6822041; doi:10.1002/ece3.5507)
Supplement: Supplementary file 4 [file ECE3-9-11545-s004.docx]

**Figure S1.** Tree based on Jetz et al. (2012) showing the phylogenetic relationships between the 145 taxa used in the study. Tips are colored by locomotor mode, showing numerous convergences in mode across taxa studied. Blue = predatory, red = flying, yellow = ground.

**Figure S2.** Individual level two-block partial least squares analysis of the bony core landmark configuration and the keratinous sheath landmark configuration using geometric morphometrics. TPS grids show differences in shape along each shape block. Blue = predatory, red = flying, yellow = ground.

Supplemental Text: Appendix S1

DESCRIPTION OF TRADITIONAL MORPHOMETRIC PROCEDURES

The claw image is imported into ImageJ. Within the program the (x, y) coordinates for each of the following points are recorded: the tip of the keratinous sheath, the tip of the bony core, the midpoint of the crescent-shaped articulation surface with the penultimate phalanx, the dorsal lip of the bony core, the dorsal lip of the keratinous sheath, and approximate midpoints along the arcs of the bony core and keratinous sheath (in text Figures 2A, 2B).

**To measure arc of bony core:**

The (x, y) coordinates of the dorsal lip of the bony core, approximate midpoint of the bony core, and tip of the bony core are used to calculate the center coordinates of the circle described by those points. This may be performed using standard formulae in Microsoft Excel or one of the many online calculation websites available (such as wolframalpha.com). To calculate the arc, equation 1 is used:

[1] 180 – 2 * DEGREES(ARCCOS((SQRT ((X_DorsalLip_ - X_TipofCore_)^2 + (Y_DorsalLip_ - Y_TipofCore_)^2)/2)/ SQRT((X_DorsalLip_ – X_CenterofCircle_)^2+( Y_DorsalLip_ - Y_CenterofCircle_)^2)))

**To measure arc of keratinous sheath:**

The (x, y) coordinates of the dorsal lip of the keratinous sheath, approximate midpoint of the keratinous sheath, and the tip of the keratinous sheath are used to calculate the center coordinates as described above. To calculate the arc, equation 2 is used:

[2] 180-2*DEGREES(ARCCOS((SQRT((X_DorsalLip_ – X_TipofSheath_)^2+(Y_DorsalLip_ - Y_TipofSheath_)^2)/2)/ SQRT((X_DorsalLip_ – X_CenterofCircle_)^2+( Y_DorsalLip_ - Y_CenterofCircle_)^2)))

**To calculate ratio of bony core to keratinous sheath:**

The (x, y) coordinates of the midpoint of the crescent-shaped articulation surface with the penultimate phalanx, tip of the bony core, and tip of the keratinous sheath are used to calculate the center coordinates as described above. The ratio is calculated using equation 3:

[3] 180-2*DEGREES(ARCCOS((SQRT((X_TipofSheath_ - X_MidpointAlongJoint_)^2+( Y_TipofSheath_ - Y_MidpointAlongJoint_)^2)/2)/ SQRT((X_TipofSheath_ – X_CenterofCircle_)^2+(Y_TipofSheath_ - Y_CenterofCircle_)^2))) / 180-2*DEGREES(ARCCOS((SQRT((X_TipofCore_ - X_MidpointAlongJoint_)^2+( Y_TipofCore_ - Y_MidpointAlongJoint_)^2)/2)/ SQRT((X_TipofSheath_ – X_CenterofCircle_)^2+( Y_TipofSheath_ - Y_CenterofCircle_)^2)))

Dividing the bony core length by total claw length describes how much of the total claw length is composed of bone versus soft tissue. For example, if the bony core length is 75 mm and the total claw length is 100 mm, then 75% of the total claw length is bone, while only 25% is made up by the keratinous sheath.

**Supplemental Table 1:** Statistical results for traditional morphometric analyses. Mean, standard deviation, and confidence intervals for intraspecific analyses. PGLS of log-transformed keratinous sheath arc and log-transformed bony core arc. Phylogenetic ANOVAs for traditional morphometrics. T-test comparing bony core and keratinous sheath log-transformed arc measurements.

**Supplemental Table 2:** Specimen number, species name, family, traditional morphometric metrics, and the first 10 PC scores for geometric morphometric data for all 580 individuals.

**Supplemental Table 3:** Statistical results for geometric morphometric analyses. Tests for allometry and phylogenetic signal. Claw shape by group including centroid size as a covariate and pairwise comparisons among ecological groups. Disparity, functional integration, and modularity analyses.

**Supplemental Table 4:** Specimen number, species name, family, centroid size mean, traditional morphometric metrics, and the first 10 PC scores for geometric morphometric data for species means (n = 145).
